# Ligand Binding Dynamics for Pre-dimerised G Protein-Coupled Receptor Homodimers: Linear Models and Analytical Solutions

**DOI:** 10.1007/s11538-017-0387-x

**Published:** 2018-01-18

**Authors:** Carla White, Lloyd J. Bridge

**Affiliations:** 1 Department of Engineering Design and Mathematics, University of the West of England, Bristol, UK; 2 Department of Mathematics, Swansea University, Swansea, UK

**Keywords:** Mathematical pharmacology, Receptor theory, G protein-coupled receptors, Ordinary differential equations

## Abstract

Evidence suggests that many G protein-coupled receptors (GPCRs) are bound together forming dimers. The implications of dimerisation for cellular signalling outcomes, and ultimately drug discovery and therapeutics, remain unclear. Consideration of ligand binding and signalling via receptor dimers is therefore required as an addition to classical receptor theory, which is largely built on assumptions of monomeric receptors. A key factor in developing theoretical models of dimer signalling is *cooperativity* across the dimer, whereby binding of a ligand to one protomer affects the binding of a ligand to the other protomer. Here, we present and analyse linear models for one-ligand and two-ligand binding dynamics at homodimerised receptors, as an essential building block in the development of dimerised receptor theory. For systems at equilibrium, we compute analytical solutions for total bound labelled ligand and derive conditions on the cooperativity factors under which multiphasic log dose–response curves are expected. This could help explain data extracted from pharmacological experiments that do not fit to the standard Hill curves that are often used in this type of analysis. For the time-dependent problems, we also obtain analytical solutions. For the single-ligand case, the construction of the analytical solution is straightforward; it is bi-exponential in time, sharing a similar structure to the well-known monomeric competition dynamics of Motulsky–Mahan. We suggest that this model is therefore practically usable by the pharmacologist towards developing insights into the potential dynamics and consequences of dimerised receptors.

## Introduction

Mathematical models have long played an important role in analytical pharmacology, which has its roots in receptor theory (Kenakin 2009; Kenakin and Williams 2014; Kenakin and Christopoulos 2011; Kenakin 2004; Colquhoun 2006). Much of this theory rests upon assumptions of equilibrium and ligands binding to monomeric receptors. It is now widely accepted that consideration of binding and signalling dynamics is an important factor in the drug discovery process (Schuetz et al. 2017). Furthermore, there is widespread acknowledgement that many receptors may exist (constitutively) as dimers or higher oligomers (Milligan 2004, 2006, 2013; Milligan and Smith 2007). Therefore, a theoretical foundation for the study of signalling via dimerised receptors is required in order to classify, quantify and simulate ligand–receptor interactions and their signalling outcomes, for both equilibrium and dynamic conditions.

G protein-coupled receptors (GPCRs) are membrane receptors of particular therapeutic importance, since they represent targets for up to approximately 50% of current drugs (Bridge et al. 2010). These receptors were long thought to exist solely as monomers, and theoretical models for GPCR signalling have largely been built around this assumption, in line with classical receptor theory for both equilibrium and dynamic models (Woodroffe et al. 2009, 2010; Bridge 2009; Bridge et al. 2010). However, it has been demonstrated and is now widely recognised that GPCRs may exist and function as dimers and higher-order oligomers (Smith and Milligan 2010; Milligan 2004, 2006; Bai 2004). The extent to which a ligand bound to one site of an oligomeric receptor complex affects ligand binding characteristics to other sites is termed cooperativity, and the effect of cross-dimer (or cross-oligomer) cooperativity on binding and signalling dynamic responses is yet to be fully elucidated theoretically.

In pursuit of potentially developing drugs which exploit ligand binding and activation crosstalk at dimeric receptors, many questions remain. For example, what are the potential advantages (to a cell or tissue) to express dimeric rather than monomeric receptors? “Expanded pharmacology” possibilities (in terms of binding and activation) may increase the diversity of potential signalling responses (via G protein coupling and signalling) towards “fine-tuning” cell responses (Smith and Milligan 2010). Identifying homodimers and heterodimers and understanding their role in signalling is a key step towards exploiting oligomerisation in *drug discovery* (Smith and Milligan 2010; Milligan 2004). The prevalence of GPCR dimers is a subject of debate, but their capacity for functional outcomes beyond those offered by monomers gives clear potential for the development of novel therapeutics (Milligan 2013). Indeed, with respect to classical receptor theory, dimerised receptors may represent “novel receptors” , i.e. receptors whose binding and signalling response profiles are more complex and offer more targetable therapeutic potential than their constituent protomers alone. Much needs to be established in terms of differential pharmacology and functional effects before dimers become tractable drug targets (Milligan 2009); development of the theory of dimerised GPCRs is therefore needed (Bai 2004), and we begin here by presenting mathematical models of ligand binding dynamics.

Mathematical modelling of binding to dimerised GPCRs has largely focused on equilibrium binding models (Casadó et al. 2007; Chidiac et al. 1997; Durroux 2005; Franco et al. 2006, 2005, 2007; Ferré et al. 2014), but the *dynamics* of ligand binding at such dimers have not been widely modelled. Typically, the equilibrium models yield analytical solutions for total bound labelled ligand, derived algebraically using mass action and receptor conservation considerations. Corresponding log dose–response (logDR) curves for bound ligand may be multiphasic, exhibiting multiple inflections (Ferré et al. 2014; Casadó et al. 2009; Rovira et al. 2009; Durroux 2005; Chidiac et al. 1997). For models of dimerised receptor binding, this departure from monophasic logDR curves (typical of monomeric receptor binding) may be quantified by a *dimer cooperativity index* (Giraldo 2008; Casadó et al. 2007; Franco et al. 2008), which relates to the apparent binding cooperativity in the Hill function sense. Within a Hill function analysis, however, there is the possibility that information is missed due to the inability of Hill coefficients to distinguish interaction mechanisms (Prinz 2010). These works appear to be the state of the art in practical GPCR models, while more mathematically abstract approaches are taken elsewhere: an algebra of dimerisation is presented in Woolf and Linderman (2004), and generalised multi-site binding models are analysed in Juška (2008).

Dynamic models of binding and signalling for dimerised receptors are less common than equilibrium models. Spatial models of the dimerisation process and subsequent signalling are developed in Mayawala et al. (2006), while dynamics of receptor and transducer protein dimerisation are studied using an ordinary differential equation (ODE) model in Vera et al. (2008), wherein it is suggested that homodimerisation may serve to regulate signalling over multiple timescales. Ligand-induced dimerisation of VEGF receptors is modelled using ODEs in Mac Gabhann and Popel (2007). In order to lay the foundations for dynamic modelling of signalling via dimerised GPCRs, we refer to a more recent study of GPCR ligand binding dynamics (May et al. 2011). Therein, linear ODE models of single-ligand and competition dynamics at pre-dimerised homodimeric receptors are presented, with a brief model analysis and numerical data fitting, without presenting analytical solutions to the ODE systems. In the current work, we describe a simplified formulation of the May et al. models and derive their analytical solutions. For the single-ligand model, the solution structure (bi-exponential in time, reflecting two distinct eigenvalues) is reminiscent of Motulsky–Mahan competition dynamics at a monomer (Motulsky and Mahan 1984).

In this paper, we develop mathematical models for the dynamics of ligand binding at pre-dimerised receptors. In Sect. 2, we formulate and solve (analytically) a linear ODE model for single-ligand (*A*) binding kinetics at constitutively dimerised receptors. We first relate our model to previous models and find an analytical equilibrium solution for total bound ligand. From this solution, we derive a condition under which multiple inflections in the logDR curve appear, in terms of the mechanistic binding cooperativity coefficient ($\alpha$). Further, we show that the time-dependent problem has an analytical solution which may be easily constructed and computed without the need for numerical ODE solvers and use this solution to simulate the binding dynamics. In Sect. 3, we extend our model to account for the presence of a second competing ligand (*B*). Again, we relate to previous models and begin by finding the equilibrium solution. This enables us to derive a condition for multiple inflections in the logDR curve for total bound ligand A, which this time depends on multiple mechanistic binding cooperativity coefficients and the concentration of *B*. The ODE model for competition dynamics is linear as before, but its analytical solution is more laborious to compute. We again simulate the binding dynamics to explore the effects of the dynamic cooperativity factors. In Sect. 4, we conclude with a discussion of our main results, underlining our contribution to the literature, and considering dynamic modelling in the context of mathematics supporting drug discovery.

## Initial Model: Single Ligand

### Model Formulation

**Fig. 1:**
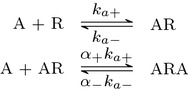
Schematic representing the reactions resulting from the binding of a single ligand

In this initial model, we consider a single-ligand, *A*, binding to a pre-dimerised receptor complex. For the purposes of this paper, we assume all dimers are homodimers and so consist of two identical receptors bound together. We denote these dimers as *R* for simplicity in the model but keep the assumption that ligand molecules can bind to either side of the dimer. Association and dissociation rates are $k_{a+}$ and $k_{a-}$, respectively, and we assume there is no bias to which protomer the ligand molecule binds to first. As the second ligand binding may be affected by one side of the dimer being already bound, we introduce $\alpha =\alpha _+/\alpha _-$ which represents the equilibrium binding cooperativity, that is the factor change in affinity for the dimer when it is already ligand bound. The value $\alpha =1$ represents neutral cooperativity, and $\alpha >1$ and $\alpha <1$ represent positive and negative cooperativity, respectively. Figure 1 shows the system of biochemical reactions. Since *R* represents a dimer, *AR* is the complex created by a single-ligand molecule binding to a protomer and *ARA* is a dual bound receptor.

### Differential Equations

Applying the law of mass action, the binding kinetics are governed by the following system of ordinary differential equations (ODEs):

1a $$\begin{aligned} \frac{\text {d}[R]}{\text {d}t}&=k_{a-}[AR]-k_{a+}[A][R], \end{aligned}$$

1b $$\begin{aligned} \frac{\text {d}[AR]}{\text {d}t}&= k_{a+}[A][R]-k_{a-}[AR] -\alpha _+k_{a+}[A][AR]+\alpha _-k_{a-}[ARA], \end{aligned}$$

1c $$\begin{aligned} \frac{\text {d}[ARA]}{\text {d}t}&=\alpha _+k_{a+}[A][AR]-\alpha _-k_{a-}[ARA], \end{aligned}$$

Conservation of receptors in the system allows us to give the total concentration of dimerised receptors as $D_\mathrm{tot}=[R]+[AR]+[ARA]$. We use this to reduce the system to two ODEs which, taking [*A*] as a constant, we write in the form of $\frac{\text {d}}{\text {d}t}\mathbf{X }(t)=M\mathbf{X }(t)+\mathbf{f }$:

2 $$\begin{aligned} \frac{\text {d}}{\text {d}t}\begin{pmatrix} [AR] \\ [ARA] \end{pmatrix}= & {} \left( \begin{array}{cc} -(k_{a-}+k_{a+}[A]+\alpha _+k_{a+}[A])&{}\quad \alpha _-k_{a-}-k_{a+}[A] \\ \alpha _+k_{a+}[A] &{}\quad -\alpha _-k_{a-} \end{array}\right) \begin{pmatrix} [AR] \\ [ARA] \end{pmatrix}\nonumber \\&+\,\begin{pmatrix} k_{a+}[A]D_{tot} \\ 0 \end{pmatrix}, \end{aligned}$$

with initial conditions

3 $$\begin{aligned} \begin{pmatrix} [AR](0) \\ [ARA](0) \end{pmatrix}=\begin{pmatrix} 0 \\ 0 \end{pmatrix}. \end{aligned}$$

### Equilibrium Analysis

In the spirit of classical receptor theory, we first investigate the equilibrium behaviour of the system, in particular the effect of the equilibrium cooperativity factor $\alpha$. The equilibrium relationships are

4a $$\begin{aligned}{}[AR]&=K_A[A][R], \end{aligned}$$

4b $$\begin{aligned}&=\alpha K_A[A][AR], \end{aligned}$$

where $K_A=k_{a+}/k_{a-}$ is the equilibrium association constant and $\alpha =\alpha _+/\alpha _-$ is the equilibrium binding cooperativity. The total concentration of dimers is

5 $$\begin{aligned} D_\mathrm{tot}&=[R]+[AR]+[ARA] \nonumber \\&=[R](1+K_A[A]+\alpha K_A^2[A]^2), \end{aligned}$$

which we can combine with equations 2.4 to express the equilibrium concentrations in terms of parameters only, giving

6a $$\begin{aligned}{}[R]&=\frac{1}{1+K_A[A]+\alpha K_A^2[A]^2}D_\mathrm{tot}, \end{aligned}$$

6b $$\begin{aligned}&=\frac{K_A[A]}{1+K_A[A]+\alpha K_A^2[A]^2}D_\mathrm{tot},\end{aligned}$$

6c $$\begin{aligned}&=\frac{\alpha K_A^2[A]^2}{1+K_A[A]+\alpha K_A^2[A]^2}D_\mathrm{tot}. \end{aligned}$$

We can clearly see that as $[A]\rightarrow \infty$ the concentrations of [*R*] and [*AR*] fall to zero, whereas [*ARA*] tends to $D_\mathrm{tot}$. The total amount of ligand bound is of primary interest, since this is often an experimentally measurable quantity (May et al. 2011). At equilibrium, we have $A_\mathrm{bound}=[AR]+2[ARA]$ due to a single-ligand molecule being bound in [*AR*] and two-ligand molecules being bound in [*ARA*]. We therefore find

7 $$\begin{aligned} A_\mathrm{bound}=\frac{(K_A[A]+2\alpha K_A^2[A]^2)}{1+K_A[A]+\alpha K_A^2[A]^2}D_\mathrm{tot}. \end{aligned}$$

This result is equivalent to the result (Giraldo 2008) states when discussing a mechanistic model for a single-ligand binding to a dimerised receptor. As the maximal ligand bound is $2D_\mathrm{tot}$, we can calculate the $EC_{50}$ value for $A_\mathrm{bound}$ (the concentration giving half-maximal effect) as

8 $$\begin{aligned} A_{50} = \frac{1}{K_A\sqrt{\alpha }}. \end{aligned}$$

**Fig. 2:**
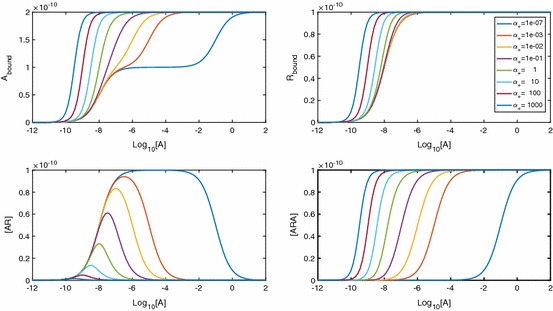
LogDR curves for $\alpha$ ranging from extreme negative to extreme positive cooperativity. Association and dissociation rates are kept at $k_{a+}=1\times 10^7\,\hbox {M}^{-1}\,\hbox {s}^{-1}$ and $k_{a-}=0.1\,\hbox {s}^{-1}$, respectively, and $D_\mathrm{tot}$ is set at $1\times 10^{-10}\,\hbox {M}$. We see that extra inflections appear in $A_\mathrm{bound}$ when we have very negative cooperativity

Figure 2 shows how the equilibrium cooperativity factor $\alpha$ affects the log dose–response (logDR) binding relationship. Increasing cooperativity leads to dimers becoming dual bound for lower concentrations of [*A*] and lower peaks in [*AR*]. Further, for $A_\mathrm{bound}$, biphasic logDR curves with multiple inflections are possible. With extreme negative cooperativity, we see three inflections in the curve instead of just one. It can be shown (“Appendix B” ) that the inflection point that appears in all curves is at the point $[A]=1/K_A\sqrt{\alpha }$, which in this case is the same value as the $EC_{50}$ due to the symmetric nature of the curves. Also, with increased $\alpha >1$, we see an approximate leftward shift in the curve.

Since the existence of extra inflections depends on $\alpha$ we seek a condition on $\alpha$ for when these appear. Following the calculation in “Appendix B,” we find that for three inflections we require

9 $$\begin{aligned} 0<\alpha <1/16. \end{aligned}$$

We note that this is different from Giraldo’s dimer cooperativity index condition for apparent cooperativity (Giraldo 2008). From a curve-fitting perspective, $\alpha =1/4$ is mechanistically negatively cooperative, but would give apparent cooperativity in the Hill function sense (see Giraldo 2008). When (9) holds, the inflection at $[A]=A_{50}$ changes from a rising inflection point to a falling one and we get two extra inflection points, one for $[A]<A_{50}$ and one for $[A]>A_{50}$ at the points

10 $$\begin{aligned}{}[A]=\frac{1-8\alpha \pm \sqrt{(16\alpha -1)(4\alpha -1)}}{2\alpha K_A}. \end{aligned}$$

We see in Fig. 2 that this results in a biphasic curve. We note here that a biphasic logDR curve with three inflections as shown would not be seen for monomeric receptors. An experimental logDR curve may be suggestive of pre-dimerised receptors and very negative cooperativity.

### Binding Dynamics–Analytical Solutions

The exact solution to the initial value problems 2, 3 may be constructed by calculating the eigenvalues of the coefficient matrix and by using the method of undetermined coefficients (“Appendix D”), or by the method of Laplace transform (as in Motulsky and Mahan 1984). Both methods give the analytical solutions as:

11 $$\begin{aligned}{}[AR](t)= & {} \frac{k_{a+}[A]D_\mathrm{tot}}{\det (M)}\left( \frac{\lambda _2(\alpha _-k_{a-}+\lambda _1)e^{\lambda _1t}-\lambda _1(\alpha _-k_{a-}+\lambda _2)e^{\lambda _2t}}{\lambda _1-\lambda _2}+\alpha _-k_{a-}\right) ,\nonumber \\ \end{aligned}$$

12 $$\begin{aligned}{}[ARA](t)= & {} \frac{\alpha _+k_{a+}^2[A]^2D_\mathrm{tot}}{\det (M)(\lambda _1-\lambda _2)}\left( \lambda _2e^{\lambda _1t}-\lambda _1e^{\lambda _2t}+\lambda _1-\lambda _2\right) , \end{aligned}$$

where the eigenvalues of the coefficient matrix *M* are

13a $$\begin{aligned} \lambda _1&=\bigg ({{\mathrm{Tr}}}(M)+\sqrt{{{\mathrm{Tr}}}(M)^2-4\det (M)}\bigg )/2, \end{aligned}$$

13b $$\begin{aligned} \lambda _2&=\bigg ({{\mathrm{Tr}}}(M)-\sqrt{{{\mathrm{Tr}}}(M)^2-4\det (M)}\bigg )/2, \end{aligned}$$

and ${{\mathrm{Tr}}}(M)$ and $\det (M)$ are the trace and determinant of *M*, respectively, given by:

14a $$\begin{aligned} {{\mathrm{Tr}}}(M)&= -\big ( (\alpha _{-}+1)k_{a-} + (\alpha _{+}+1)k_{a+}[A] \big ) \; <0, \end{aligned}$$

14b $$\begin{aligned} \det (M)&= \alpha _{-}k_{a-}^{2}+\alpha _{-}k_{a-}k_{a+}A+ \alpha _{+}(k_{a+}[A])^{2} \nonumber \\&= \alpha _{-}k_{a-}^{2}\big (1+K_{A}[A]+\alpha K_{A}^{2}[A]^{2} \big ) \; > 0, \end{aligned}$$

Our response of primary interest is the total concentration of ligand bound, $A_\mathrm{bound}$, which is given by15The solution has two exponential components, meaning we would expect to see bi-exponential time courses in general. It can be shown (“Appendix C”) that not only are eigenvalues $\lambda _1$ and $\lambda _2$ real and distinct but they are also always negative, since all parameters in the model are positive. Further, $\lambda _{1}$ gives the slow component of the dynamics, while $\lambda _{2}$ gives the fast component. Clearly, as $t \rightarrow \infty$, the exponential components will decay to zero and we recover the equilibrium concentrations as

16 $$\begin{aligned}{}[AR] = \frac{\alpha _-k_{a+}k_{a-}[A]D_\mathrm{tot}}{\det (M)},\qquad [ARA] = \frac{\alpha _+k_{a+}^2[A]^2D_\mathrm{tot}}{\det (M)}, \end{aligned}$$

giving the total ligand bound at equilibrium as

17 $$\begin{aligned} A_\mathrm{bound}=\frac{k_{a+}[A]D_\mathrm{tot}}{\det (M)}\bigg (\alpha _-k_{a-}+2\alpha _+k_{a+}[A]\bigg ), \end{aligned}$$

equivalent to (2.6).

Having analytical solutions for the dynamics allows time courses to be plotted without the need for numerical ODE solvers. This is particularly useful for pharmacologists without numerics expertise, allowing them to construct exact solutions for any parameter values in software packages such as Microsoft Excel or GraphPad Prism (accepted). In this respect, the dynamics for our dimer binding model have the same analytical structure as Motulsky–Mahan dynamics (Motulsky and Mahan 1984). In “Appendix H,” we show a sample MATLAB code for construction of the analytical solution.

### Results

**Fig. 3:**
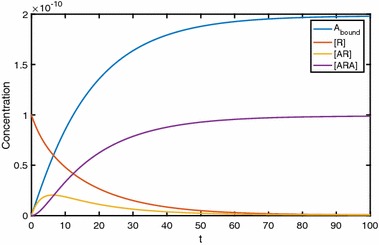
The binding of ligand *A* to a pre-dimerised receptor results in a surge in [*AR*], while positive cooperativity leads to most dimers becoming dual bound. Parameter values are $k_{a+}=1\times 10^7\,\hbox {M}^{-1}\,\hbox {s}^{-1}$ and $k_{a-}=0.1\,\hbox {s}^{-1}$ for association and dissociation rates while keeping [*A*] constant at $1\times 10^{-8}\,\hbox {M}$. Also $\alpha _+=2$ and $\alpha _-=0.01$, giving positive cooperativity, and $D_\mathrm{tot}=1\times 10^{-10}\,\hbox {M}$

**Fig. 4:**
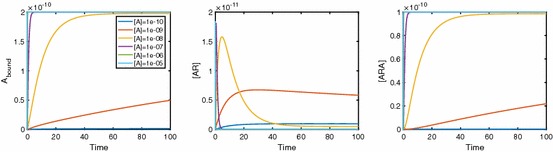
Binding dynamics with varying [*A*] in the system. With high levels of [*A*], we no longer see peaks in [*AR*]. Here, $k_{a+}=1\times 10^7\,\hbox {M}^{-1}\,\hbox {s}^{-1}$ and $k_{a-}=0.1\,\hbox {s}^{-1}, \alpha _+=2$ and $\alpha _-=0.01$ with $D_\mathrm{tot}=1\times 10^{-10}\,\hbox {M}$

Here we present numerical results which demonstrate the effects of cooperative dynamics across dimerised receptors. Figure 3 shows the binding of ligand *A* to the dimerised receptor [*R*] with the ligand being added as a constant quantity. Initially, ligand molecules bind singly to dimers, creating an increase in [*AR*]. As time increases, a positive cooperativity factor means that the chance of a second ligand molecule binding to the singularly bound dimer is increased; thus, [*ARA*] increases. This increase in [*ARA*] also has the effect of reducing [*AR*] which then falls towards zero. Hence, we see a peak in [*AR*]. In fact, this peak becomes a point of interest as we move on to look at Fig. 4. In this, we consider a range of values of [*A*] and study the effect this has on the binding dynamics. It is clear that not only does $A_\mathrm{bound}$ increase to a higher equilibrium concentration as [*A*] increases, but the equilibration timescale is shorter. For low [*A*], [*AR*] apparently approaches equilibrium monotonically, whereas a peak in [*AR*](*t*) occurs for high concentrations of *A*. The time at which a possible local extremum occurs is

18 $$\begin{aligned} t=-\,\frac{\log \left( \frac{\alpha _-k_{a-}+\lambda _1}{\alpha _-k_{a-}+\lambda _2}\right) }{\lambda _1-\lambda _2}. \end{aligned}$$

For a peak in [*AR*](*t*) (see “Appendix E”), we require

19 $$\begin{aligned} k_{a+}[A]>\alpha _-k_{a-}, \end{aligned}$$

and the corresponding peak concentration of [*AR*] is

20 $$\begin{aligned}{}[AR]_{\text {peak}}=\frac{k_{a+}[A]D_\mathrm{tot}}{\det (M)}\left( -(\alpha _-k_{a-}+\lambda _1)\left( \frac{\alpha _-k_{a-}+\lambda _1}{\alpha _-k_{a-}+\lambda _2}\right) ^{\frac{\lambda _1}{\lambda _2-\lambda _1}}+\alpha _-k_{a-}\right) .\nonumber \\ \end{aligned}$$

**Fig. 5:**
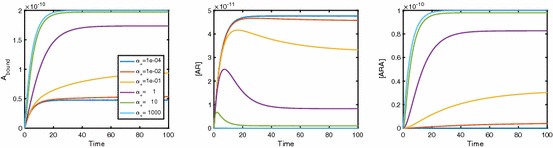
Binding dynamics with varying $\alpha$. As we move from positive to negative cooperativity, we see a more pronounced peak in [*AR*] with $A_\mathrm{bound}$ tending to lower concentrations. Here, $k_{a+}=1\times 10^7\,\hbox {M}^{-1}\,\hbox {s}^{-1}$ and $k_{a-}=0.01\,\hbox {s}^{-1}$, $[A] = 1 \times 10^{-8}\,\hbox {M}, D_\mathrm{tot}=1\times 10^{-10}\,\hbox {M}$. We fix $\alpha _- = 1$ so cooperativity varies via $\alpha _+$

In Fig. 5, we study the effect of $\alpha$ on $A_\mathrm{bound}(t)$. Clearly, the binding rate for a second ligand to the dimer increases and decreases for positive and negative cooperativity, respectively. We see a peak in [*AR*](*t*) whose timing and concentration are dependent on $\alpha _+$. As $\alpha _+$ increases, the peak in [*AR*] decreases and occurs earlier. Both [*ARA*] and $A_\mathrm{bound}$ increase as $\alpha _+$ increases.

## Two-Ligand System

### Model Formulation

We now introduce a second ligand, *B*, to the system. The rationale for this is that quantification of effects of unlabelled ligands can be achieved by competition experiments with labelled and unlabelled ligands, as seen in previous studies involving monomeric and dimeric receptors (Motulsky and Mahan 1984; May et al. 2011; Durroux 2005; Franco et al. 2006). The kinetics of this system are key in highlighting and quantifying allosteric interactions across dimerised receptors, as indicated by May et al. (2011), who discuss the influence of an unlabelled ligand on the dissociation (washout) kinetics of a labelled ligand, when dimers are present.

**Fig. 6:**
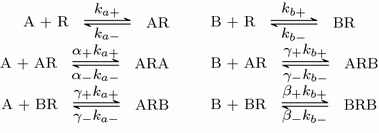
Schematic showing the binding possibilities with two ligands in the system

We denote $\beta =\beta _+/\beta _-$ as the influence a protomer bound by ligand *B* has on a second *B* molecule binding, and $\gamma =\gamma _+/\gamma _-$ as the cooperativity factor describing the interaction between *A* and *B* bound receptors. This extended system gives a set of six reactions, as can be seen in Fig. 6. Taken together, these reactions correspond to a single face of the model schematic developed by Franco et al. (2006).

### Differential Equations

As the total concentration of receptors is given by

21 $$\begin{aligned} D_\mathrm{tot}=[R]+[AR]+[BR]+[ARA]+[ARB]+[BRB], \end{aligned}$$

we may eliminate [*R*] to give a system of five linear ODEs:

22a $$\begin{aligned} \frac{\text {d}[AR]}{\text {d}t}&=-\,(k_{a+}[A]+k_{a-}+\alpha _+k_{a+}[A]-\gamma _+k_{b+}[B])[AR] -k_{a+}[A][BR]\nonumber \\&\quad \, +\,(\alpha _-k_{a-}-k_{a+}[A])[ARA]+(\gamma _-k_{b-}-k_{a+}[A])[ARB]\nonumber \\&\quad \, -\,k_{a+}[A][BRB]+k_{a+}[A]D_\mathrm{tot}, \end{aligned}$$

22b $$\begin{aligned} \frac{\text {d}[BR]}{\text {d}t}&=-\,k_{b+}[B][AR]-(k_{b+}[B]+k_{b-}+\gamma _+k_{b+}[A]+\beta _+k_{b+}[B])[BR]\nonumber \\&\quad \,-\,k_{b+}[B][ARA]+(\gamma _-k_{b-}-k_{b+}[B])[ARB]\nonumber \\&\quad \,+\,(\beta _-k_{b-}-k_{b+}[B])[BRB]+k_{b+}[B]D_\mathrm{tot},\end{aligned}$$

22c $$\begin{aligned} \frac{\text {d}[ARA]}{\text {d}t}&=\alpha _+k_{a+}[A][AR]-\alpha _-k_{a-}[ARA],\end{aligned}$$

22d $$\begin{aligned} \frac{\text {d}[ARB]}{\text {d}t}&=\gamma _+k_{b+}[B][AR]+\gamma _+k_{a+}[A][BR]\nonumber \\&\quad \, -\,\gamma _-k_{b-}[ARB]-\gamma _-k_{a-}[ARB],\end{aligned}$$

22e $$\begin{aligned} \frac{\text {d}[BRB]}{\text {d}t}&=\beta _+k_{b+}[B][BR]-\beta _-k_{b-}[BRB]. \end{aligned}$$

with initial conditions

23 $$\begin{aligned} \begin{pmatrix} [AR](0) \\ [BR](0) \\ [ARA](0) \\ [ARB](0) \\ [BRB](0) \end{pmatrix}=\begin{pmatrix} 0 \\ 0 \\ 0 \\ 0 \\ 0 \end{pmatrix}. \end{aligned}$$

We note that this system is a simplification of the one developed by May et al. (2011) (see “Appendix A”).

### Equilibrium Analysis

Once the system reaches equilibrium, we have the set of relations

24a $$\begin{aligned}{}[AR]&=K_A[A][R],\qquad [BR] =K_B[B][R],\end{aligned}$$

24b $$\begin{aligned}&=\alpha K_A^2[A]^2[R],\qquad [ARB] =\gamma K_AK_B[A][B][R],\nonumber \\ [BRB]&=\beta K_B^2[B]^2[R]. \end{aligned}$$

The receptor conservation law therefore gives that

25 $$\begin{aligned} D_\mathrm{tot}= & {} [R](1+K_A[A]+K_B[B]+\alpha K_A^2[A]^2\nonumber \\&+\,\gamma K_AK_B[A][B]+\beta K_B^2[B]^2). \end{aligned}$$

Here we consider, as in May et al. (2011), competition experiments where ligand *A* is labelled and ligand *B* is unlabelled. We can clearly see that, providing [*B*] is fixed,

26 $$\begin{aligned}{}[R],[AR],[BR],[ARB],[BRB]\rightarrow 0 \text {, and } [ARA]\rightarrow D_\mathrm{tot} \text { as } [A]\rightarrow \infty . \end{aligned}$$

The total concentration of bound ligand *A* (assumed an experimentally measurable quantity) is

27 $$\begin{aligned} A_\mathrm{bound}=[AR]+2[ARA]+[ARB], \end{aligned}$$

which, using Eqs. (3.4) and (25), gives

28 $$\begin{aligned} A_\mathrm{bound}&=\frac{K_A[A]+2\alpha K_A^2[A]^2+\gamma K_AK_B[A][B]}{1+K_A[A]+K_B[B]+\alpha K_A^2[A]^2+\gamma K_AK_B[A][B]+\beta K_B^2[B]^2}D_\mathrm{tot}. \end{aligned}$$

**Fig. 7:**
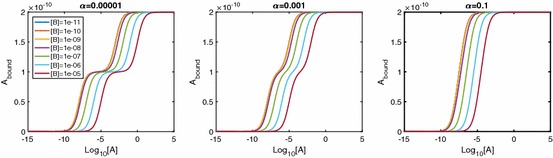
LogDR curve for varying $\alpha _{+}$ shows extra inflections when we have low A-A cooperativity regardless of [*B*]. Plot parameters are $k_{a+}=k_{b+}=1\times 10^7\,\hbox {M}^{-1}\,\hbox {s}^{-1}, k_{a-}=k_{b-}=0.1\,\hbox {s}^{-1}, [A]=1\times 10^{-8} \hbox {M}, D_\mathrm{tot}=1\times 10^{-10}\,\hbox {M}$. All other cooperativity values are set to 1

**Fig. 8:**
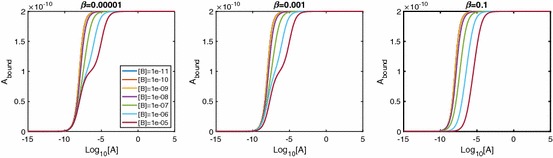
LogDR curve for varying $\beta _{+}$ shows extra inflections appear when we have both low B-B cooperativity and low [*B*]. Plot parameters are $k_{a+}=k_{b+}=1\times 10^7\,\hbox {M}^{-1}\,\hbox {s}^{-1}, k_{a-}=k_{b-}=0.1\,\hbox {s}^{-1}, [A]=1\times 10^{-8}\,\hbox {M}, D_\mathrm{tot}=1\times 10^{-10}\,\hbox {M}$. All other cooperativity values are set to 1

**Fig. 9:**
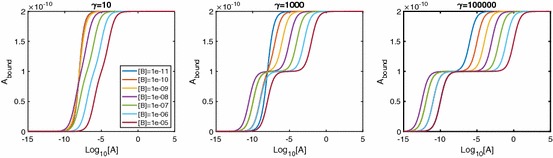
LogDR curve for varying $\gamma _{+}$ shows extra inflections when we have high A-B cooperativity as well as low [*B*]. Plot parameters are $k_{a+}=k_{b+}=1\times 10^7\,\hbox {M}^{-1}\,\hbox {s}^{-1}, k_{a-}=k_{b-}=0.1\,\hbox {s}^{-1}, [A]=1\times 10^{-8}\,\hbox {M}, D_\mathrm{tot}=1\times 10^{-10}\,\hbox {M}$. All other cooperativity values are set to 1

As the maximal $A_\mathrm{bound}$ for varying [*A*] remains as $2D_\mathrm{tot}$, we calculate the $EC_{50}$ for $A_\mathrm{bound}$ as

29 $$\begin{aligned} A_{50}=\frac{1}{K_A}\sqrt{\frac{1+K_B[B]+\beta K_B^2[B]^2}{\alpha }}, \end{aligned}$$

which we note is independent of the A-B cooperativity factor $\gamma$. In Figs. 7, 8, 9, we show the effects of each of the cooperativity factors $\alpha , \beta , \gamma$ in turn. In each case, we plot logDR curves for $A_\mathrm{bound}$ for a range of values for [*B*].

In Fig. 7, we consider a range of values of $\alpha$, fixing $\beta$ and $\gamma$. Again we see extra inflections for low values of $\alpha$, similarly to the single-ligand system. While molecules of *B* bind to *AR* to form *ARB* complexes, such a low $\alpha$ means that very few molecules of *A* bind to *AR*; thus, [*ARA*] remains low, unless [*A*] is sufficiently large. For $[A]\rightarrow \infty$, both [*AR*] and $[ARB]\rightarrow 0$ regardless of $\alpha$, giving local maxima in the logDR curves for these two variables (see individual species plots in Fig. 17). Very small $\alpha$ requires very large [*A*] to effect this downturn in [*AR*] and [*ARB*], giving plateaus at the maximal response in the logDR for [*AR*] and [*ARB*]. It is these plateaus that change the nature of the original inflection point for $A_\mathrm{bound}$, as well as creating two extra inflections.

**Fig. 10:**
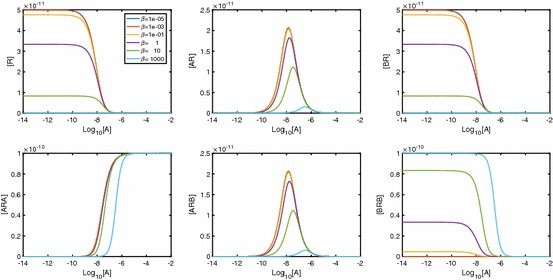
Individual species plots for a varying $\beta$. Plots were created with parameters $K_A=K_B=10^8\,\hbox {M}^{-1}, \alpha =\gamma =1, [B]=10^{-5}\,\hbox {M}, D_\mathrm{tot}=10^{-10}\,\hbox {M}$

Figure 8 shows the effect of varying the B-B cooperativity factor, $\beta$. We see extra inflections appearing for conditions of both low $\beta$ and high [*B*]. For high [*B*] and small $\beta <1$, a relatively large proportion of receptor will be in [*BR*] complexes, unless [*A*] is sufficiently large. Again, corresponding logDR curves for [*AR*] and [*ARB*] exhibit local maxima (see Fig. 10).

**Fig. 11:**
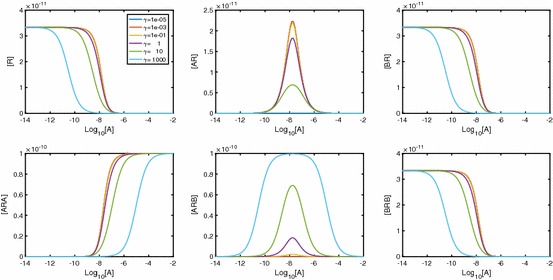
Individual species plots for a varying $\gamma$. Plots were created with parameters $K_A=K_B=10^8\,\hbox {M}^{-1}, \alpha =\beta =1, [B]=10^{-8}\,\hbox {M}, D_\mathrm{tot}=10^{-10}\,\hbox {M}$

In Fig. 9, we show the effects of A-B cooperativity $\gamma$ and see that, for extra inflections, $\gamma$ is required to be high as opposed to low. We again require there to be a high concentration of *B* in the system. The individual species curves in Fig. 11 again show the requirement of sufficiently large [*A*] for a downturn in [*AR*] and [*ARB*], but this time with the largest [*ARB*] values corresponding to large $\gamma$.

Investigating these extra inflections in $A_\mathrm{bound}$ (“Appendix G”), we find that there is always an inflection point at

30 $$\begin{aligned}{}[A]=\frac{1}{K_A}\sqrt{\frac{1+K_B[B]+\beta K_B^2[B]^2}{\alpha }}. \end{aligned}$$

We further find that extra inflections appear under the condition

31 $$\begin{aligned} 16\alpha (1+K_B[B]+\beta K_B^2[B]^2)<(\gamma K_B[B]+1)^2. \end{aligned}$$

Again we have an extra inflection at either side of the original one, at the points

32 $$\begin{aligned}{}[A]=\frac{m^2-8\alpha n\pm \sqrt{(m^2-16\alpha n)(m^2-4\alpha n)}}{2\alpha K_A m}, \end{aligned}$$

where $$m=1+\gamma K_B[B]$$ and $$n=1+K_B[B]+\beta K_B^2[B]^2$$.

### Time Course Results

Analytical solutions for the system (3.2) are theoretically possible, given that the system is linear. However, the task of computing eigenvalues exactly becomes laborious and impractical; we instead construct the solutions by numerical evaluation of the eigenvalues. A numerical ODE solver could also be used. Here we present time course simulations for two-ligand competition.

**Fig. 12:**
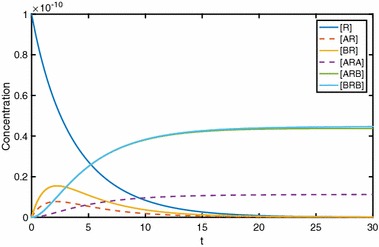
In the time course plot, we see peaks in both [*AR*] and [*BR*]. Parameter values: $k_{a+}=k_{b+}=1\times 10^7\,\hbox {M} ^{-1}\,\hbox {s}^{-1}, k_{a-}=k_{b-}=0.1\,\hbox {s}^{-1}, \alpha _+=\beta _+=\gamma _+=2, \alpha _-=\beta _-=\gamma _-=0.01, [A]=1\times 10^{-8}\,\hbox {M}, [B]=2\times 10^{-8}\,\hbox {M}, D_\mathrm{tot}=1\times 10^{-10}\,\hbox {M}$

In Fig. 12, we show time courses for each receptor state, for conditions of positive A-A, A-B and B-B cooperativities. We note [*AR*] and [*ARA*] dynamics similar to the single-ligand problem. Initially, a single-ligand molecule binds to one of the free receptors in the dimer, increasing [*AR*] before the positive cooperativity factor soon after binds a second ligand molecule, thus increasing [*ARA*] and lowering [*AR*].

**Fig. 13:**
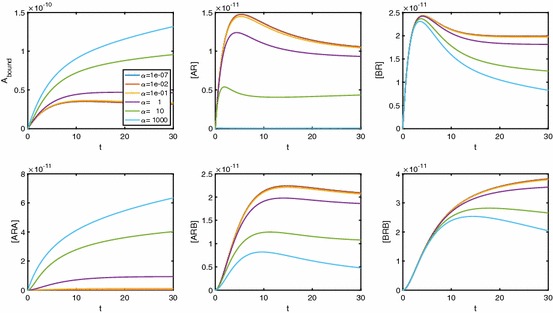
As $\alpha _{+}$ increases, the peak in [*AR*] decreases, while the peak in [*BR*] becomes more pronounced. Parameter values: $k_{a+}=k_{b+}=1\times 10^7\,\hbox {M}^{-1}\,\hbox {s}^{-1}, k_{a-}=k_{b-}=0.1\,\hbox {s}^{-1}, [A]=1\times 10^{-8}\,\hbox {M}, [B]=2\times 10^{-8}\,\hbox {M}, D_\mathrm{tot}=1\times 10^{-10}\,\hbox {M}$. All other cooperativity factors are set to 1

In Fig. 13, we see the effect $\alpha$ has on the time course dynamics of the system. We look at a range of values of $\alpha$ while keeping $\beta$ and $\gamma$ fixed, giving neutral cooperativity. It is clear that regardless of the A-A cooperativity, we get peaks in both [*AR*] and [*BR*]. As $\alpha$ increases, the peak in [*BR*] is lower and the peak in [*AR*] is later and higher. As $\alpha$ increases, we also see that [*ARA*] increases and both [*ARB*] and [*BRB*] decrease. Further, peaks are apparent in [*ARB*] and, for large $\alpha$, in [*BRB*].

**Fig. 14:**
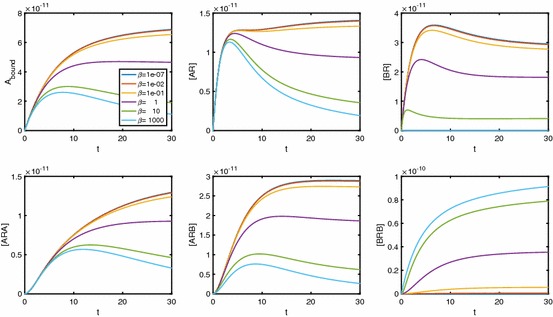
As $\beta$ increases, the peak in [*BR*] decreases, while the peak in [*AR*] becomes more pronounced. Parameter values: $k_{a+}=k_{b+}=1\times 10^7\,\hbox {M}^{-1}\,\hbox {s}^{-1}, k_{a-}=k_{b-}=0.1\,\hbox {s}^{-1}, [A]=1\times 10^{-8}, [B]=2\times 10^{-8}, D_\mathrm{tot}=1\times 10^{-10}\,\hbox {M}$. All other cooperativity factors are set to 1 so cooperativity is neutral for A-A and A-B and B-B cooperativity depends solely on $\beta _+$

In Fig. 14, we vary cooperativity factor $\beta$. Non-monotonic behaviour is clearly shown for all receptor states except [*BRB*], and the potential for non-monotonic signal $A_\mathrm{bound}$ is clearly demonstrated.

**Fig. 15:**
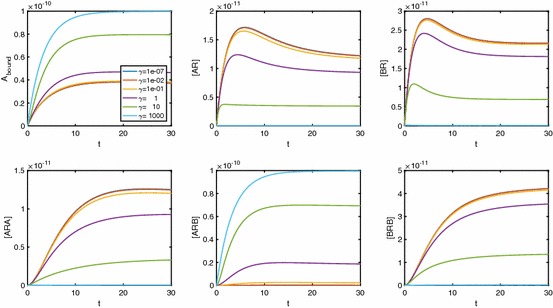
As $\gamma$ increases, the peak in both [*AR*] and [*BR*] decreases. Parameter values: $k_{a+}=k_{b+}=1\times 10^7\,\hbox {M}^{-1}\,\hbox {s}^{-1}, k_{a-}=k_{b-}=0.1\,\hbox {s}^{-1}, [A]=1\times 10^{-8}\,\hbox {M}, [B]=2\times 10^{-8}\,\hbox {M}, D_\mathrm{tot}=1\times 10^{-10}\,\hbox {M}$. All other cooperativity factors are set to 1 so cooperativity is neutral for A-A and B-B and A-B cooperativity depends solely on $\gamma _+$

In Fig. 15, we demonstrate the effect of varying $\gamma$. As molecules of *A* and *B* become bound to free receptors, we again see [*AR*] and [*BR*] increase. With small $\gamma$, a second *A* or *B* molecule causes [*ARA*] and [*BRB*] to increase, while [*ARB*] remains low. As $\gamma$ increases past neutral cooperativity, we begin to see instead that it is the alternate ligand that binds to singularly bound receptors, meaning that [*ARB*] increases, while [*ARA*] and [*BRB*] remain low.

## Discussion

In this paper, we have presented dynamic models of ligand binding to pre-dimerised GPCR homodimers, for both a single-ligand and two-ligand competition. The models are linear ODE systems, allowing analytical solutions for time-dependent and equilibrium responses. The model formulation, solution and results serve as a contribution to the field of pharmacological modelling and are expected to be of practical use, given the ease with which we can compute solutions. In particular, the bi-exponential single-ligand binding kinetics show a similar model solution structure to the widely used Motulsky–Mahan model for competition binding at monomers (Motulsky and Mahan 1984). We therefore propose that this model can be adopted, interpreted and implemented with relative ease by pharmacologists, and as such, we have provided a recipe for computational solution.

Time course computation for our models is straightforward, given the analytical solutions we have developed. However, there are interesting features in the equilibrium logDR curves for the experimental readout (signal) $A_\mathrm{bound}$. We have noted the possibility for multiple inflections in logDR curves, for both single- and two-ligand binding. Multiphasic features in logDR curves are not reproducible by standard Hill functions (Veroli et al. 2015) which only support single inflection curves. For single-ligand binding, multiphasic logDR curves theoretically rule out monomeric receptor binding as the only ligand binding mechanism; such experimental data therefore would suggest another binding setup, possibly due to dimeric receptors. The existence of multiple inflections depends on the level of *cooperativity* across a dimerised receptor, and importantly we are able to give a condition on the single-ligand cooperativity factor for a three-inflection curve (9). The practical use of this condition is clearly in assessing the sign and magnitude of cooperativity towards quantitative classification of drug–receptor interactions. Given that Hill functions are not suitable for fully characterising multiphasic logDR curves (Veroli et al. 2015), our analysis here goes beyond dimer cooperativity indices which stem in part from cooperativity in the Hill function sense (Giraldo 2008); the present work concerns *mechanistic cooperativity* which is explicit in the original model schematic, as opposed to empirical measures from a more limited model. In Veroli et al. (2015), it is noted that multiphasic logDR curves have particular importance in a number of contexts including cancer pharmacology, and effort should be made to move beyond Hill function fitting wherever possible.

Two-ligand competition at GPCR homodimers has previously been discussed and modelled (May et al. 2011). In addition to the therapeutic relevance of agonist–antagonist competition (May et al. 2011; Bridge et al. 2010), for homodimeric receptors, dissociation kinetic assays may represent a more sensitive forum for detecting negative cooperativity than logDR analysis alone. As such, we have reduced the model of May et al. (2011) and outlined the analytical solution structure for the time-dependent problem. Further, considering a system with a labelled ligand *A* and an unlabelled ligand *B*, we are able to find conditions on [*B*] and the three cross-dimer cooperativity factors for multiple inflections in the logDR curve for $A_\mathrm{bound}$ (Eq. 31), which again is designed to aid interpretation of experimental data and clear identification of parameter regimes which may have therapeutic significance.

Our simple models of ligands binding to constitutive homodimers, and particularly the explicit statement and elucidation of analytical results, represent an important addition to receptor theory. We remark that this should be considered only the foundation of the theory for binding and activation dynamics of GPCR dimers. There is a clear pathway for building on this foundation to expand upon this model towards simulating and understanding the potential diversity binding and signalling outcomes of non-monomeric receptors. In particular, the following natural extensions and considerations should be explored in future work.

**Fig. 16:**
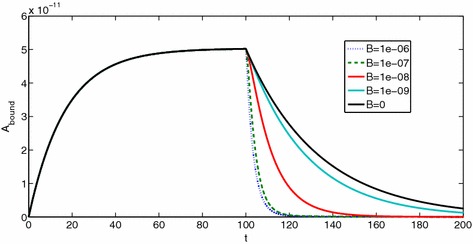
Binding then washout experiments, for labelled ligand *A* and unlabelled ligand *B*, varying [*B*]. Firstly binding of *A* in the absence of *B*, then dissociation of *A*, with *A* being washed out, with varying concentrations of *B*. The dissociation curves depend on [*B*], indicating cooperativity across the dimer (such a result would not be seen for monomeric receptor). Parameter values: $k_{a+}=1\times 10^7\,\hbox {M}^{-1}\,\hbox {s}^{-1}$, $k_{a-}=0.03\,\hbox {s}^{-1}, [A]=3\times 10^{-9}$M, $k_{b+}=1\times 10^7 \,\hbox {M}^{-1}\,\hbox {s}^{-1}, k_{b-}=0.01\,\hbox {s}^{-1}, \alpha _{+}=0.005, \alpha _{-}=1, \beta _{+}=1, \beta _{-}=10, \gamma _{+}=1, \gamma _{-}=10$. All three equilibrium cooperativities are negative, in qualitative agreement with (May et al. 2011)

**Fig. 17:**
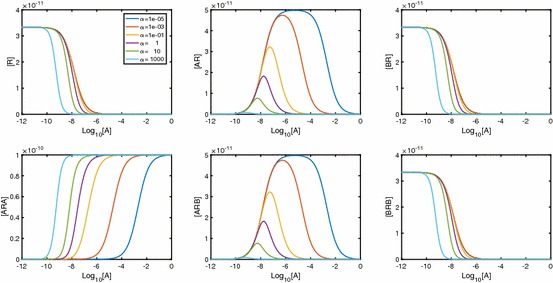
Individual species plots for a varying $\alpha$. Plots were created with parameters $K_A=K_B=1\times 10^8\,\hbox {M}^{-1}/\hbox {s}_{-1}, \beta =\gamma =1, [B]=10^{-8}\,\hbox {M}, D_\mathrm{tot}=10^{-10}$

1. Heterodimeric GPCRs are widely acknowledged to exist (Smith and Milligan 2010; Milligan 2004, 2006, 2009). Here, we have reduced the model of May et al. (2011) using the symmetry inherent in their homodimer model, resulting in a clean formulation amenable to algebraic manipulation. Extension to a more general model which will encompass heterodimers will require reversion to the May et al. schematic, but with an implicit asymmetry which will allow for an “extra level of complexity” (Bai 2004).
2. Higher-order complexes may exist (Smith and Milligan 2010; Juška 2008), and while a general functional signalling model for an $n\mathrm{th}$-order oligomer may be impractical, a ligand binding model of the type developed in the current work should be possible and may be valuable in distinguishing between orders of oligomer.
3. Here, we have assumed that all dimeric receptors are constitutively produced, are the only receptor entities and have not considered the dimerisation process itself. However, a mixture of monomers, dimers and higher oligomers may exist (Smith and Milligan 2010; Milligan 2004), and ligand-induced effects on the dimerisation process have also been noted for GPCRs (Smith and Milligan 2010; Bai 2004) and other receptors (Mac Gabhann and Popel 2007). Therefore, we propose to expand the model to include constitutive and ligand-induced dimerisation dynamics. This will allow theoretical investigation of questions put forth in Milligan (2004) regarding the effects of fraction of receptors which are dimers, dynamics and lifetime of dimerisation and the effect of ligands. Further modelling in this direction will draw on dimerisation models (Vera et al. 2008; Mac Gabhann and Popel 2007), towards a general, hybrid model for constitutive and ligand-induced dimers and ligand binding. Further complexity may be possible within a dimer population, whereby only a fraction of dimers may support cross-dimer crosstalk (Durroux 2005).
4. In keeping with classical analytical pharmacology approaches, we have first considered models for ligand binding; the cooperativity factors $\alpha , \beta , \gamma$ are *binding cooperativities*. A diverse range of pharmacological *effects* is theoretically possible from dimeric receptors, in terms of receptor activation and signalling dynamics (Milligan 2004). Much is yet to be understood about signalling downstream of dimerised receptors, and how to distinguish between ligand–receptor level crosstalk and downstream crosstalk when interpreting experimental data (Milligan 2006, 2009; Chabre et al. 2009). A natural extension of our model will include receptor activation (Giraldo 2008), G protein binding (Milligan 2009; Bai 2004), and ultimately G protein activation and cycling (Woodroffe et al. 2010; Bridge 2009). In moving towards simulating downstream functional effects, new modelling studies will require consideration of cross-dimer cooperativity in (i) ligand binding, (ii) receptor activation and (iii) G protein binding.
5. Beyond simulating and using our theoretical results to identify parameter *ranges*, our models will be useful for parameter estimation to return best-fit parameter values to experimental data, as in May et al. (2011). Considering time course data for ligand binding, and possibly washout experiments, it is important to determine which of the model’s unknown kinetic parameters are theoretically identifiable from the given readout. In addition to model fitting with any available data and pseudo-experimental data, using methods such as genetic algorithms (Ashyraliyev et al. 2009), an important (but often overlooked) computation in bio-modelling is that of structural identifiability analysis (SIA). We propose to investigate the identifiability properties of our linear models using Laplace transform methods (Godfrey 1986). While beyond our scope here, this is an important avenue of investigation, given that in May et al. (2011), an initial parameterisation of the model therein suggests a non-identifiability issue; estimated equilibrium cooperativities are reported rather than all individual kinetic constants. The analysis in May et al. (2011) further suggests that a combination of association and washout experiments will be useful in estimating kinetic parameters relating to dimerised receptor binding. The simplified models presented in the current work will be used to simulate such experiments, and a detailed SIA will be performed towards parameterising ligand–dimer interactions. In Fig. 16, we show an initial simulation of binding followed by washout, in qualitative agreement with the results presented in May et al. (2011).

The current work provides a theoretical foundation for the study of ligands binding dimerised receptors. We consider this theory as a vital step towards the potential exploitation of dimerised receptors in a therapeutic and drug discovery context. Understanding the functional, physiological and therapeutic significance of receptor dimers is an ongoing challenge (Smith and Milligan 2010), with some questions still outstanding over the possible requirement of dimerisation for receptor function (Milligan 2004). Given the broad appreciation of GPCR dimerisation, it is now widely accepted that dimers are true drug receptors, and cross-dimer binding and activation cooperativity gives rise to a “new kind of pharmacological target” (Ferré et al. 2014) whose allosteric cooperativity is key. So-called dual molecules can be used in targeting dopamine–adenosine heterodimers in the treatment of Parkinson’s disease, while it is suggested that opioid–cannabinoid heterodimers could be targeted for pain relief (Franco et al. 2008). Mathematical modelling will undoubtedly continue to be a key tool in simulating and quantifying in vitro ligand–receptor interactions and towards understanding of in vivo dynamics. In view of the potential functional diversity of dimerised receptors, the further development of dimerisation dynamics as a field of receptor theory is a crucial mathematical challenge towards drug discovery.

## Simplification of the May et al. Model

System (3.2) is seen to be a simplification of the model developed by May et al. (2011), equivalent by taking

33 $$\begin{aligned} k_{a+}&=\frac{{\widetilde{k}}_{a+}}{2},\quad k_{a-}={\widetilde{k}}_{a-}\quad k_{b+}=\frac{{\widetilde{k}}_{b+}}{2},\quad k_{b-}={\widetilde{k}}_{b-}, \end{aligned}$$

34 $$\begin{aligned} \alpha _+&=2{\widetilde{\alpha }}_+,\quad \alpha _-=\frac{{\widetilde{\alpha }}_-}{2},\quad \beta _+=2{\widetilde{\beta }}_+, \nonumber \\ \beta _-&=\frac{{\widetilde{\beta }}_-}{2},\quad \gamma _+=2{\widetilde{\gamma }}_+,\quad \gamma _-=\frac{{\widetilde{\gamma }}_-}{2}, \end{aligned}$$

noting that

35 $$\begin{aligned}{}[AR]=\frac{\widetilde{[AR]}}{2},\quad [BR]=\frac{\widetilde{[BR]}}{2},\quad [ARB]=\frac{\widetilde{[ARB]}}{2}, \end{aligned}$$

where a tilde denotes a parameter or variable in the May et al. (2011) model.

## Extra Inflections in the Single-Ligand $A_\mathrm{bound}$ Curve

In Fig. 2, we saw extra inflections in the logDR curve for $A_\mathrm{bound}$. We investigate these and derive the condition given in Eq. (9). Writing $A_\mathrm{bound}$ in terms of $X=K_A[A]$ for simplification, we have

36 $$\begin{aligned} A_\mathrm{bound}=\frac{X+2\alpha X^2}{1+X+\alpha X^2}D_\mathrm{tot}. \end{aligned}$$

To investigate the inflections, we require $\frac{\text {d}^2A_\mathrm{bound}}{\text {d}Y^2}$ where

37 $$\begin{aligned} Y=\log _{10}[A]=\frac{\ln \big (\frac{X}{K_A}\big )}{\ln {10}}, \end{aligned}$$

(and $X=K_A10^Y$). Using Di Bruno’s Johnson (2002) formula for the chain rule for second derivatives, we find

38 $$\begin{aligned} \frac{\text {d}^2A_\mathrm{bound}}{\text {d}Y^2}=\left( \frac{\text {d}X}{\text {d}Y}\right) ^2.\frac{\text {d}^2A_\mathrm{bound}}{\text {d}X^2}+\frac{\text {d}^2X}{\text {d}Y^2}.\frac{\text {d}A^{bound}}{\text {d}X}. \end{aligned}$$

Calculating each term individually, we have

39 $$\begin{aligned} \frac{\text {d}X}{\text {d}Y}= & {} K_A10^Y\ln {10}=X\ln {10}, \end{aligned}$$

40 $$\begin{aligned} \frac{\text {d}^2X}{\text {d}Y^2}= & {} K_A10^Y(\ln {10})^2=X(\ln {10})^2, \end{aligned}$$

41 $$\begin{aligned} \frac{\text {d}A_\mathrm{bound}}{\text {d}X}= & {} \frac{1+4\alpha X+\alpha X^2}{(1+X+\alpha X^2)^2}D_\mathrm{tot}, \end{aligned}$$

42 $$\begin{aligned} \frac{\text {d}^2A_\mathrm{bound}}{\text {d}X^2}= & {} \frac{-2(1-2\alpha +3\alpha X+6\alpha ^2 X^2+\alpha ^2 X^3)}{(1+X+\alpha X^2)^3}D_\mathrm{tot}. \end{aligned}$$

Substituting into (38), and after some simplification, we find

43 $$\begin{aligned} \frac{\text {d}^2A_\mathrm{bound}}{\text {d}Y^2}=\frac{X(\ln {10})^2(1+(8\alpha -1)X+(\alpha -8\alpha ^2)X^3-\alpha ^2 X^4)}{(1+X+\alpha X^2)^3}D_\mathrm{tot}. \end{aligned}$$

To locate the possible inflection points, we find zeros of $d^2A_\mathrm{bound}/dY^2$, which occur when

44 $$\begin{aligned} X(1+(8\alpha -1)X+(\alpha -8\alpha ^2)X^3-\alpha ^2 X^4)&=0, \nonumber \\ \Rightarrow \qquad -X(\alpha X^2-1)(1-X+8\alpha X+\alpha X^2)&=0. \end{aligned}$$

This gives the trivial solution at $X=0$ and solutions at

45 $$\begin{aligned} X=\pm \,\frac{1}{\sqrt{\alpha }}. \end{aligned}$$

However, we are only interested in positive solutions, so we conclude that we always have an inflection point at $X=1/\sqrt{\alpha }$, that is when $[A]=1/K_A\sqrt{\alpha }$. We note that this is the same concentration of [*A*] that gives half the maximal response. Other possible inflections occur for roots $X>0$ of the quadratic equation

46 $$\begin{aligned} 1-X+8\alpha X+\alpha X^2=0, \end{aligned}$$

which gives solutions at

47 $$\begin{aligned} X_\pm&=\frac{1-8\alpha \pm \sqrt{(8\alpha -1)^2-4\alpha }}{2\alpha }\nonumber \\&=\frac{1-8\alpha \pm \sqrt{(16\alpha -1)(4\alpha -1)}}{2\alpha }. \end{aligned}$$

We see that we get a repeated root if

48 $$\begin{aligned} (16\alpha -1)(4\alpha -1)=0, \end{aligned}$$

that is when $\alpha =1/16$ or $\alpha =1/4$. However, we see that the root is only positive for $\alpha =1/16$. To get real and distinct solutions, we require

49 $$\begin{aligned} (16\alpha -1)(4\alpha -1)>0, \end{aligned}$$

that is when $\alpha >\frac{1}{4}$ or when $\alpha <\frac{1}{16}$. We also need to confirm whether the roots are positive or negative under these conditions. So first noting that

50 $$\begin{aligned}&(1-8\alpha )^2-4\alpha >0, \end{aligned}$$

51 $$\begin{aligned}&\Rightarrow (1-8\alpha )^2>4\alpha , \end{aligned}$$

52 $$\begin{aligned}&\Rightarrow \sqrt{(1-8\alpha )^2-4\alpha }<8\alpha -1. \end{aligned}$$

Now if $\alpha <1/16$, then $1-8\alpha>1-1/2=1/2>0$; thus, we have two positive roots under this condition. However, if $\alpha >1/4$, then $1-8\alpha<1-2=-\,1<0$; hence, under this condition we get two negative roots. So we conclude that we get two possible extra inflection points under the condition $0<\alpha <1/16$.

To confirm that these are inflection points and classify the type of inflection, we consider $\text {d}^3A_\mathrm{bound}/\text {d}Y^3$. Using the Di Bruno formula (see Johnson (2002)) for higher derivatives, or MATLAB’s Symbolic Toolbox, we find:

53

For the $EC_{50}$ value, at $X=1/\sqrt{\alpha }$, and substituting this into the third derivative. After some simplification, we have

54 $$\begin{aligned} -\frac{(\ln {10})^3(8\alpha -2\sqrt{\alpha })}{(2\sqrt{\alpha }+1)^2}D_\mathrm{tot}, \end{aligned}$$

which we can see is positive for any $0<\alpha <1/16$ and negative for any $\alpha >1/16$.

Similarly for the extra inflections, we found at $X=\tau _1$ and $X=\tau _2$ when $\alpha <1/16$. Substituting both of these in results in a value of

55 $$\begin{aligned} \frac{(\ln {10})^3(16\alpha -1)}{8(4\alpha -1)^2}D_\mathrm{tot}, \end{aligned}$$

which clearly gives a negative result for any $0<\alpha <1/16$.

Looking at these results, we can see that if $\alpha >1/16$, we have the single inflection point, which in this case has a concavity that changes from convex to concave. However, when $0<\alpha <1/16$, this inflection point reverses and so is concave changing to convex. In this case, we also get the two extra points, with each of those being convex changing to concave at the point.

## Confirming the Eigenvalues of the Coefficient Matrix are Real, Distinct and Negative

Before we can solve the system of equations to give analytical solutions, we need to know whether the eigenvalues, given in Eq. (2.13), of the coefficient matrix *M* are real and distinct so we can determine the form of the general solution. Being able to state whether these are also positive or negative allows us to analyse these solutions. The eigenvalues are defined as

56 $$\begin{aligned} \lambda _{1,2}= & {} \frac{{{\mathrm{Tr}}}(M)\pm \sqrt{{{\mathrm{Tr}}}(M)^2-4\det (M)}}{2}\nonumber \\= & {} \frac{{{\mathrm{Tr}}}(M)\pm \sqrt{{{\mathrm{Tr}}}(M)^2-4\det (M)}}{2}, \end{aligned}$$

so we can see that the discriminant is

57 $$\begin{aligned} D={{\mathrm{Tr}}}(M)^2-4\det (M). \end{aligned}$$

Expanding *D*, we have

58 $$\begin{aligned} D&={{\mathrm{Tr}}}(M)^2-4\det (M), \nonumber \\&=(\alpha _--1)^2k_{a-}^2-2(\alpha _--1)(\alpha _++1)k_{a+}k_{a-}[A]\nonumber \\&\quad +\,(\alpha _++1)^2k_{a+}^2[A]^2-4\alpha _+k_{a+}^2[A]^2+4\alpha _+\alpha _-k_{a+}k_{a-}[A], \nonumber \\&=(\alpha _--1)^2k_{a-}^2-2(\alpha _+\alpha _-+\alpha _--\alpha _+-1)k_{a+}k_{a-}[A]\nonumber \\&\quad +\,(\alpha _++1)^2k_{a+}^2[A]^2-4\alpha _+k_{a+}^2[A]^2+4\alpha _+\alpha _-k_{a+}k_{a-}[A], \nonumber \\&=(\alpha _--1)^2k_{a-}^2-2(\alpha _+\alpha _-+\alpha _--\alpha _+-1)k_{a+}k_{a-}[A]\nonumber \\&\quad +\,(\alpha _+-1)^2k_{a+}^2[A]^2+4\alpha _+\alpha _-k_{a+}k_{a-}[A], \nonumber \\&=(\alpha _--1)^2k_{a-}^2+2(\alpha _+\alpha _--\alpha _-+\alpha _++1)k_{a+}k_{a-}[A]\nonumber \\&\quad +\,(\alpha _+-1)^2k_{a+}^2[A]^2, \nonumber \\&=(\alpha _--1)^2k_{a-}^2+2((\alpha _--1)(\alpha _+-1)+2\alpha _+)k_{a+}k_{a-}[A]\nonumber \\&\quad +\,(\alpha _+-1)^2k_{a+}^2[A]^2, \nonumber \\&=\big ((\alpha _--1)k_{a-}+(\alpha _+-1)k_{a+}[A]\big )^2\nonumber \\&\quad +\,4\alpha _+k_{a+}k_{a-}[A], \nonumber \\&>0. \end{aligned}$$

Hence, *D* is always positive, providing that all parameters are positive.

We can also show that both $\lambda _1$ and $\lambda _2$ are always negative. First noting that ${{\mathrm{Tr}}}(M)$ is negative due to all terms being negative, and

59 $$\begin{aligned} {{\mathrm{Tr}}}(M)^2-4\det (M)&>0 \end{aligned}$$

60 $$\begin{aligned} \Rightarrow {{\mathrm{Tr}}}(M)^2&>4\det (M) \end{aligned}$$

61 $$\begin{aligned} \Rightarrow \sqrt{{{\mathrm{Tr}}}(M)^2-4\det (M)}&<{{\mathrm{Tr}}}(M). \end{aligned}$$

Thus, it is clear that both ${{\mathrm{Tr}}}(M)+\sqrt{{{\mathrm{Tr}}}(M)^2-4\det (M)}<0$ and ${{\mathrm{Tr}}}(M)-\sqrt{{{\mathrm{Tr}}}(M)^2-4\det (M)}<0$; hence, both eigenvalues are negative.

## Analytical Solutions for the Single-Ligand Dynamics

One of the main motivations for this paper is to be able to give exact solutions to the system, allowing for simulations to be run easily; hence, in this appendix we derive these solutions as stated in Eqs. (11) and (12). To find a solution to the system, we first find the eigenvalues of the coefficient matrix, *M*.

62 $$\begin{aligned} \lambda _{1,2}=\frac{{{\mathrm{Tr}}}(M)\pm \sqrt{{{\mathrm{Tr}}}(M)^2-4\det (M)}}{2}. \end{aligned}$$

The eigenvectors corresponding to eigenvalues $\lambda _1$ and $\lambda _2$ are

63 $$\begin{aligned} v_1=\begin{pmatrix} \lambda _1+\alpha _-k_{a-} \\ \alpha _+k_{a+}[A] \end{pmatrix},\qquad v_2=\begin{pmatrix} \lambda _2+\alpha _-k_{a-} \\ \alpha _+k_{a+}[A] \end{pmatrix}. \end{aligned}$$

Thus, we can state that a solution to the system will be of the form

64 $$\begin{aligned} \mathbf{X }(t)=c_1\mathbf{v }_1e^{\lambda _1t}+c_2\mathbf{v }_2e^{\lambda _2t}+\mathbf{X }_p(t), \end{aligned}$$

where $\mathbf{X }_p(t)$ is a particular solution, found by solving $M\mathbf{X }+\mathbf{f }=\mathbf{0 }$, thus

65 $$\begin{aligned} \mathbf{X }^p=\frac{k_{a+}[A]D_\mathrm{tot}}{\det (M)}\begin{pmatrix} \alpha _-k_{a-} \\ \alpha _+k_{a+}[A] \end{pmatrix}. \end{aligned}$$

As $t\rightarrow \infty$, all other terms in the solution tend to zero, due to $\lambda _{1,2}$ always being negative; thus, the particular solution tells us what [*AR*] and [*ARA*] will be at equilibrium. We now use the initial conditions to solve the system and find the coefficients $c_1$ and $c_2$, as

66 $$\begin{aligned} \mathbf{C } = \begin{pmatrix} c_{1} \\ c_{2} \end{pmatrix} = \frac{k_{a+}[A]D_\mathrm{tot}}{\det (M)(\lambda _1-\lambda _2)}\begin{pmatrix} \lambda _2 \\ -\lambda _1 \end{pmatrix}. \end{aligned}$$

We find the solutions

67 $$\begin{aligned}{}[AR](t)= & {} \frac{k_{a+}[A]D_\mathrm{tot}}{\det (M)}\left( \frac{\lambda _2(\alpha _-k_{a-}+\lambda _1)e^{\lambda _1t}-\lambda _1(\alpha _-k_{a-}+\lambda _2)e^{\lambda _2t}}{\lambda _1-\lambda _2}+\alpha _-k_{a-}\right) ,\nonumber \\ \end{aligned}$$

68 $$\begin{aligned} (t)= & {} \frac{\alpha _+k_{a+}^2[A]^2D_\mathrm{tot}}{\det (M)(\lambda _1-\lambda _2)}\left( \lambda _2e^{\lambda _1t}-\lambda _1e^{\lambda _2t}+\lambda _1-\lambda _2\right) . \end{aligned}$$

## A Condition for Existence of a Peak in [*AR*](*t*)

As we saw in Figs. 3, 4, we sometimes get a peak in [*AR*](*t*). In this appendix, we investigate these peaks to find a condition Eq. (19) for peak existence and calculate this peak concentration Eq. (20) is. The peak time is given by Eq. (18)

69 $$\begin{aligned} t=-\,\frac{\ln \left( \frac{\alpha _-k_{a-}+\lambda _1}{\alpha _-k_{a-}+\lambda _2}\right) }{\lambda _1-\lambda _2}. \end{aligned}$$

For peak existence, we therefore require

70 $$\begin{aligned} \frac{\alpha _-k_{a-}+\lambda _1}{\alpha _-k_{a-}+\lambda _2}>0, \end{aligned}$$

where $\lambda _{2}<\lambda _{1}<0$. Hence, we require, for a positive peak time,

$$\begin{aligned} 0< \frac{\alpha _-k_{a-}+\lambda _1}{\alpha _-k_{a-}+\lambda _2} <1, \end{aligned}$$

which requires that

$$\begin{aligned} \alpha _{-}k_{a-}+\lambda _{1}<0. \end{aligned}$$

Since $\lambda _{1}=\frac{-(\alpha _{-}+1)k_{a-}-(\alpha _{+}+1)k_{a+}[A] + \sqrt{D}}{2}$, where *D* is the discriminant for the coefficient matrix, we find our requirement to be

$$\begin{aligned} 0< (\alpha _{+}+1)k_{a+}[A]-(\alpha _{-}+1)k_{a-} < \sqrt{D}, \end{aligned}$$

which eventually gives

71 $$\begin{aligned} \alpha _{-}<K_{A}[A]. \end{aligned}$$

To evaluate the peak concentration [*AR*], we take Eq. (18)

72 $$\begin{aligned} t=-\,\frac{\log \left( \frac{\alpha _-k_{a-}+\lambda _1}{\alpha _-k_{a-}+\lambda _2}\right) }{\lambda _1-\lambda _2}. \end{aligned}$$

and substituting the peak time into Eq. (11), giving

73 $$\begin{aligned}&\frac{k_{a+}[A]D_\mathrm{tot}}{\det (M)}\left( \frac{\lambda _2(\alpha _-k_{a-}+\lambda _1)\left( \frac{\alpha _-k_{a-}+\lambda _1}{\alpha _-k_{a-}+\lambda _2}\right) ^{\frac{\lambda _1}{\lambda _2-\lambda _1}}-\lambda _1(\alpha _-k_{a-}+\lambda _2)\left( \frac{\alpha _-k_{a-}+\lambda _1}{\alpha _-k_{a-}+\lambda _2}\right) ^{\frac{\lambda _2}{\lambda _2-\lambda _1}}}{\lambda _1-\lambda _2}+\alpha _-k_{a-}\right) \end{aligned}$$

74 $$\begin{aligned}&\quad =\frac{k_{a+}[A]D_\mathrm{tot}}{\det (M)}\left( \frac{\left( \lambda _2(\alpha _-k_{a-}+\lambda _1)-\left( \frac{\alpha _-k_{a-}+\lambda _1}{\alpha _-k_{a-}+\lambda _2}\right) \lambda _1(\alpha _-k_{a-}+\lambda _2)\right) \left( \frac{\alpha _-k_{a-}+\lambda _1}{\alpha _-k_{a-}+\lambda _2}\right) ^{\frac{\lambda _1}{\lambda _2-\lambda _1}}}{\lambda _1-\lambda _2}+\alpha _-k_{a-}\right) \end{aligned}$$

75 $$\begin{aligned}&\quad =\frac{k_{a+}[A]D_\mathrm{tot}}{\det (M)}\left( \frac{\left( \lambda _2(\alpha _-k_{a-}+\lambda _1)-\lambda _1(\alpha _-k_{a-}+\lambda _1)\right) \left( \frac{\alpha _-k_{a-}+\lambda _1}{\alpha _-k_{a-}+\lambda _2}\right) ^{\frac{\lambda _1}{\lambda _2-\lambda _1}}}{\lambda _1-\lambda _2}+\alpha _-k_{a-}\right) \end{aligned}$$

76 $$\begin{aligned}&\quad =\frac{k_{a+}[A]D_\mathrm{tot}}{\det (M)}\left( \frac{(\lambda _2-\lambda _1)(\alpha _-k_{a-}+\lambda _1)\left( \frac{\alpha _-k_{a-}+\lambda _1}{\alpha _-k_{a-}+\lambda _2}\right) ^{\frac{\lambda _1}{\lambda _2-\lambda _1}}}{\lambda _1-\lambda _2}+\alpha _-k_{a-}\right) \end{aligned}$$

77 $$\begin{aligned}&\quad =\frac{k_{a+}[A]D_\mathrm{tot}}{\det (M)}\left( -(\alpha _-k_{a-}+\lambda _1)\left( \frac{\alpha _-k_{a-}+\lambda _1}{\alpha _-k_{a-}+\lambda _2}\right) ^{\frac{\lambda _1}{\lambda _2-\lambda _1}}+\alpha _-k_{a-}\right) \end{aligned}$$

as the corresponding concentration of [*AR*].

## Individual Species Dose–Response Curves

To further help us understand the behaviour observed in the logDR curves for $A_\mathrm{bound}$, we look to the individual species plots. In Fig. 17, we see plateaus in the logDR curves of [*AR*] and [*ARA*] when we have very low A-A cooperativity. These are the main contributions to the inflections in $A_\mathrm{bound}$.

## Investigating the Extra Inflections in the Two-Ligand $A_\mathrm{bound}$ curve

Similarly to the single-ligand system, we also get extra inflections in the $A_\mathrm{bound}$ curve when we have two ligands in the system. In this appendix, we investigate these to derive the condition given in Eq. (31) under which we get these extra inflections. We begin by writing $A_\mathrm{bound}$ as

78 $$\begin{aligned} A_\mathrm{bound}=\frac{X+2\alpha X^2+\gamma XY}{1+X+Y+\alpha X^2 +\gamma XY +\beta Y^2}D_\mathrm{tot}, \end{aligned}$$

where $X=K_A[A]$ and $Y=K_B[B]$. For possible inflections, we first calculate $d^2A_\mathrm{bound}/dZ^2$ where $Z=\log _{10}[A]=\ln (\frac{X}{K_A})/\ln {10}$. We find

79

80 $$\begin{aligned}&=\frac{X(\ln {10})^2(n-\alpha X^2)(mn+(8\alpha n-m^2)X+\alpha mX^2)}{(n+mX+\alpha X^2)^3} \end{aligned}$$

where $m=1+\gamma Y$ and $n=1+Y+\beta Y^2$. Clearly, we have the trivial root at $X=0$ as well as a root when

81 $$\begin{aligned} n-\alpha X^{2}=0, \end{aligned}$$

that is when

82 $$\begin{aligned} X=\pm \,\sqrt{\frac{n}{\alpha }}. \end{aligned}$$

As we are only interested in positive roots, we conclude that we have a possible inflection at

83 $$\begin{aligned} X_c=\sqrt{\frac{n}{\alpha }}, \end{aligned}$$

which corresponds to the $EC_{50}$ concentration. We also get extra roots when

84 $$\begin{aligned} mn+(8\alpha n-m^2)X+\alpha mX^2=0, \end{aligned}$$

which using the quadratic formula gives us

85 $$\begin{aligned} X_{\pm }&=\frac{m^2-8\alpha n\pm \sqrt{(8\alpha -m^2)^2-4\alpha m^2n}}{2\alpha m}\end{aligned}$$

86 $$\begin{aligned}&=\frac{m^2-8\alpha n\pm \sqrt{(m^2-16\alpha n)(m^2-4\alpha n)}}{2\alpha m}. \end{aligned}$$

We see that we get a single, real root when

87 $$\begin{aligned} (m^2-16\alpha n)(m^2-4\alpha n)=0, \end{aligned}$$

that is when $m^2=16\alpha n$ or $m^2=4\alpha n$. However, we see that if $m^2=4\alpha n$, then $X=-\,2n/m$, hence a negative root. Whereas if $m^2=16\alpha n$, then $X=4n/m$, so we have a positive root. Thus, we can conclude that we have a possible inflection at $X=4n/m$ under the condition of $m^2=16\alpha n$.

Finally, we have two real distinct roots when

88 $$\begin{aligned} (m^2-16\alpha n)(m^2-4\alpha n)>0. \end{aligned}$$

As $m^2-16\alpha n<m^2-4\alpha n$, then this holds if either $m^2>16\alpha n$ or $m^2<4\alpha n$. To confirm whether these conditions give positive or negative roots, we first note that

89 $$\begin{aligned} (m^2-8\alpha n)^2-4\alpha m^2n&>0\end{aligned}$$

90 $$\begin{aligned} \Rightarrow (m^2-8\alpha n)^2&>4\alpha m^2n\end{aligned}$$

91 $$\begin{aligned} \Rightarrow \sqrt{(m^2-8\alpha n)^2-4\alpha m^2n}&<m^2-8\alpha n. \end{aligned}$$

Using this, we can state that if $m^2>16\alpha n$, then $m^2-8\alpha n>16\alpha n-8\alpha n=8\alpha n>0$; hence, we have two positive roots. Conversely, if $m^2<4\alpha n$, then $m^2-8\alpha n<4\alpha n-8\alpha n=-\,4\alpha n<0$; hence, both roots are negative. Thus, we can conclude that we get two positive roots, and possible inflection points, under the condition $m^2>16\alpha n$. That is

92 $$\begin{aligned} (1+\gamma Y)^2>16\alpha (1+Y+\beta Y^2). \end{aligned}$$

To confirm that these are in fact inflection points, we first confirm that $X_-<X_c<X_+$. First noting that $m^2-16\alpha n<m^2-4\alpha n\Rightarrow (m^2-16\alpha n)^2<(m^2-16\alpha n)(m^2-4\alpha n)\Rightarrow m^2-16\alpha n<\sqrt{(m^2-16\alpha n)(m^2-4\alpha n)}$, then we can state that

93 $$\begin{aligned} X_-&=\frac{m^2-8\alpha n-\sqrt{(m^2-16\alpha n)(m^2-4\alpha n)}}{2\alpha m} \end{aligned}$$

94 $$\begin{aligned}&<\frac{m^2-8\alpha n-(m^2-16\alpha n)}{2\alpha m}\end{aligned}$$

95 $$\begin{aligned}&=\frac{8\alpha n}{2\alpha m}\end{aligned}$$

96 $$\begin{aligned}&=\frac{4n}{m}\end{aligned}$$

97 $$\begin{aligned}&=\sqrt{\frac{16n^2}{m^2}}\end{aligned}$$

98 $$\begin{aligned}&<\sqrt{\frac{n}{\alpha }}=X_c. \end{aligned}$$

Thus, we can confirm that $X_-<X_c$. Also

99 $$\begin{aligned} X_+&=\frac{m^2-8\alpha n+\sqrt{(m^2-16\alpha n)(m^2-4\alpha n)}}{2\alpha m}\end{aligned}$$

100 $$\begin{aligned}&>\frac{m^2-8\alpha n}{2\alpha m}\end{aligned}$$

101 $$\begin{aligned}&=\sqrt{\frac{(m^2-8\alpha n)^2}{4\alpha ^2m^2}}\end{aligned}$$

102 $$\begin{aligned}&>\sqrt{\frac{4\alpha m^2n}{4\alpha ^2m^2}}\end{aligned}$$

103 $$\begin{aligned}&=\sqrt{\frac{n}{\alpha }}=X_c, \end{aligned}$$

hence confirming that $X_c<X_+$. We now use the second derivative to confirm there is a sign change at each of the points. We can see as $X\rightarrow 0^{+}$, we have $d^2A_\mathrm{bound}/dZ^2 > 0$. Taking ${\hat{X}}_-=4n/m$ as a point between $X_-$ and $X_c$, we evaluate

104 $$\begin{aligned} \left. \frac{\text {d}^2A_\mathrm{bound}}{\text {d}Z^2}\right| _{X={\hat{X}}_{-}} = -\frac{12n^3(m^2-16\alpha n)^2}{m^4}<0; \end{aligned}$$

thus, we can confirm there is an inflection point at $X_-$ with the curve changing from convex to concave. Taking ${\hat{X}}_+=(m^2-8\alpha n)/2\alpha m$ as a point between $X_c$ and $X_+$, we evaluate

105 $$\begin{aligned} \left. \frac{\text {d}^2A_\mathrm{bound}}{\text {d}Z^2}\right| _{X={\hat{X}}_{-}} = \frac{(m^2-8\alpha n)((m^2-8\alpha n)^2-4\alpha m^2n)^2}{32\alpha ^3m^4}>0; \end{aligned}$$

hence, there is a sign change at the point $X_c$ with the curve changing from convex to concave. Finally, taking the limit as $X\rightarrow \infty$, we see that $d^2A_\mathrm{bound}/dZ^2 < 0$; thus, we have a third inflection point with the curve changing from convex to concave. So to conclude, under the conditions $16\alpha (1+Y+\beta Y^2)<(1+\gamma Y)^2$, we have three inflection points.
